# Supplementary material for: Effect of mRNA Delivery Modality and Formulation on Cutaneous mRNA Distribution and Downstream eGFP Expression
Source: Pharmaceutics. 2022 Jan 8;14(1):151. doi: 10.3390/pharmaceutics14010151 (PMC8780332; doi:10.3390/pharmaceutics14010151)
Supplement: Supplementary file 1 [file pharmaceutics-14-00151-s001.zip › File S1.pdf]

Supplementary Materials: Effect of mRNA Delivery Modality and Formulation on Cutaneous mRNA Distribution and Downstream eGFP Expression

Aditya R. Darade, Maria Lapteva, Thomas Hoffmann, Markus Mandler, Achim Schneeberger and Yogeshvar N. Kalia

FILE S1

Table S1 : Site of mRNA delivery and extent of GFP expression by the different skin cells

Delivery technique	Delivery site	Types of cells expressing GFP		
		Keratinocytes	Fibroblasts / Vascular endothelium	Appendageal epithelium
**Intradermal injection**	Dermis	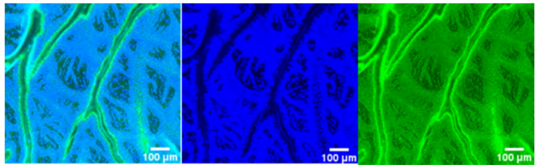	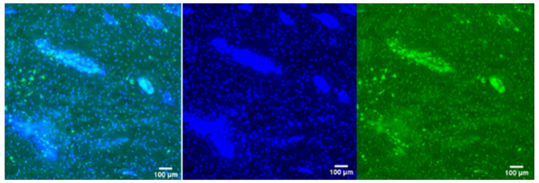	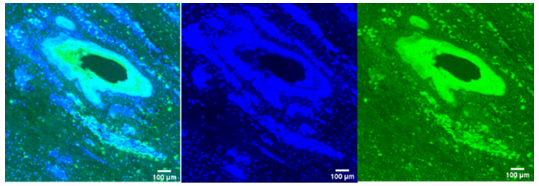
**Nanopass injection**	Dermis	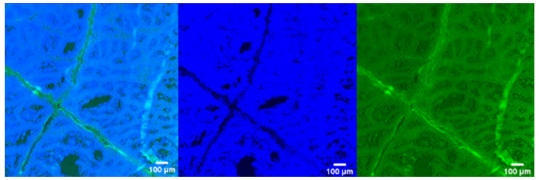	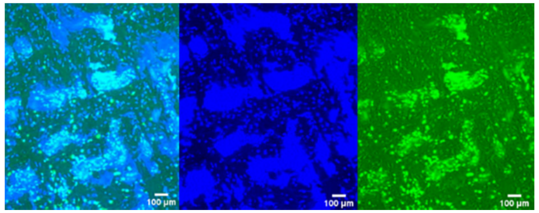	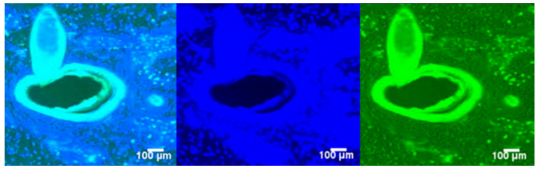
**Dermojet**	Dermis	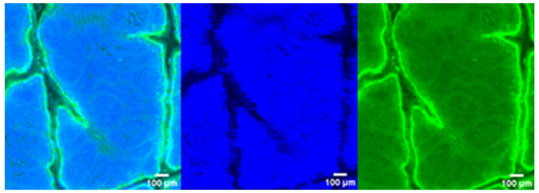	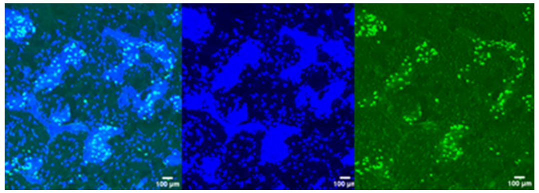	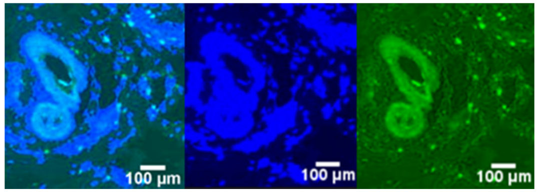
**Laser**	Epidermis	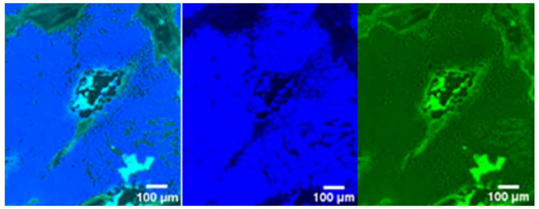	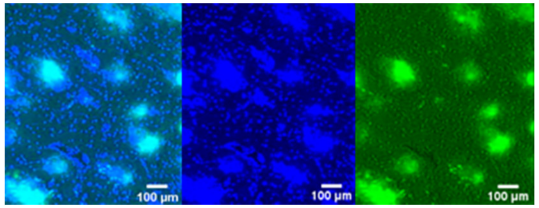	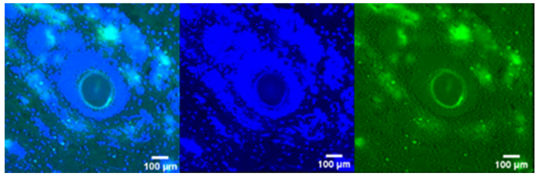
