# Supplementary figures and images for: Effect of mRNA Delivery Modality and Formulation on Cutaneous mRNA Distribution and Downstream eGFP Expression
Source: Pharmaceutics. 2022 Jan 8;14(1):151. doi: 10.3390/pharmaceutics14010151 (PMC8780332; doi:10.3390/pharmaceutics14010151)

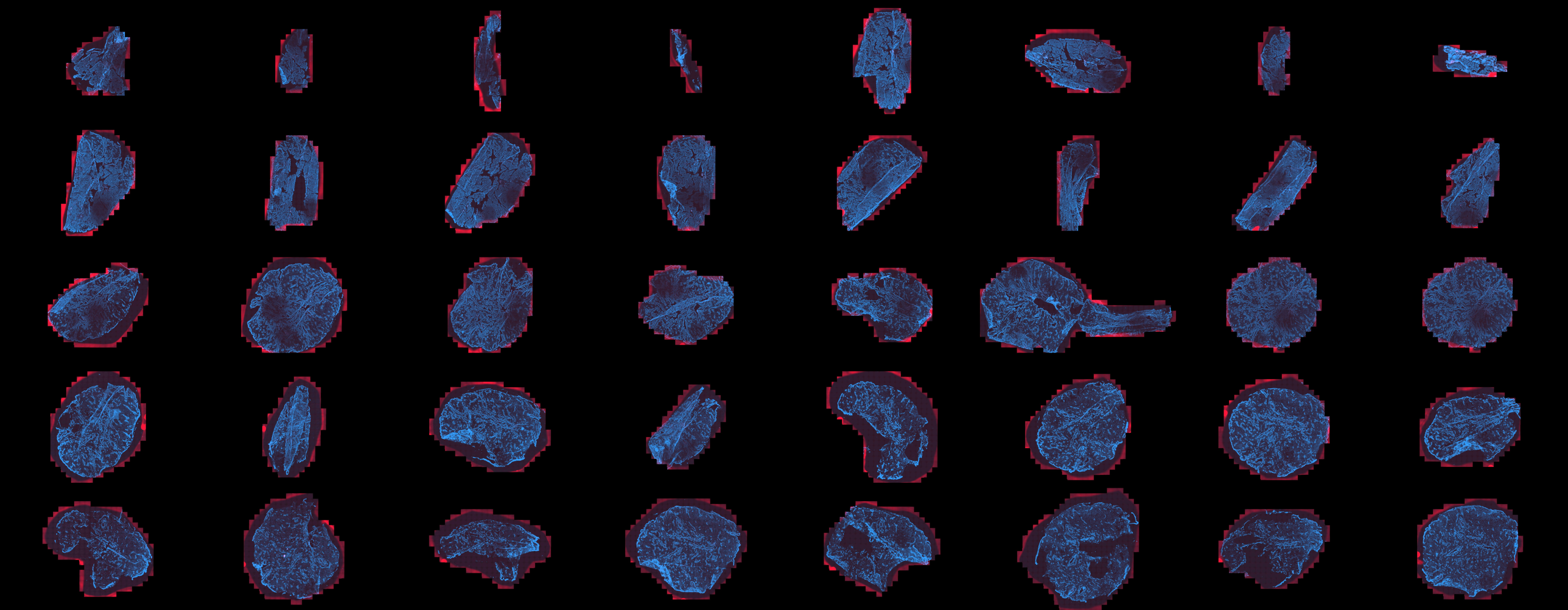

Supplement: Supplementary file 1 [file pharmaceutics-14-00151-s001.zip › File S2 - all samples images/1 - untreated cy5.jpg]

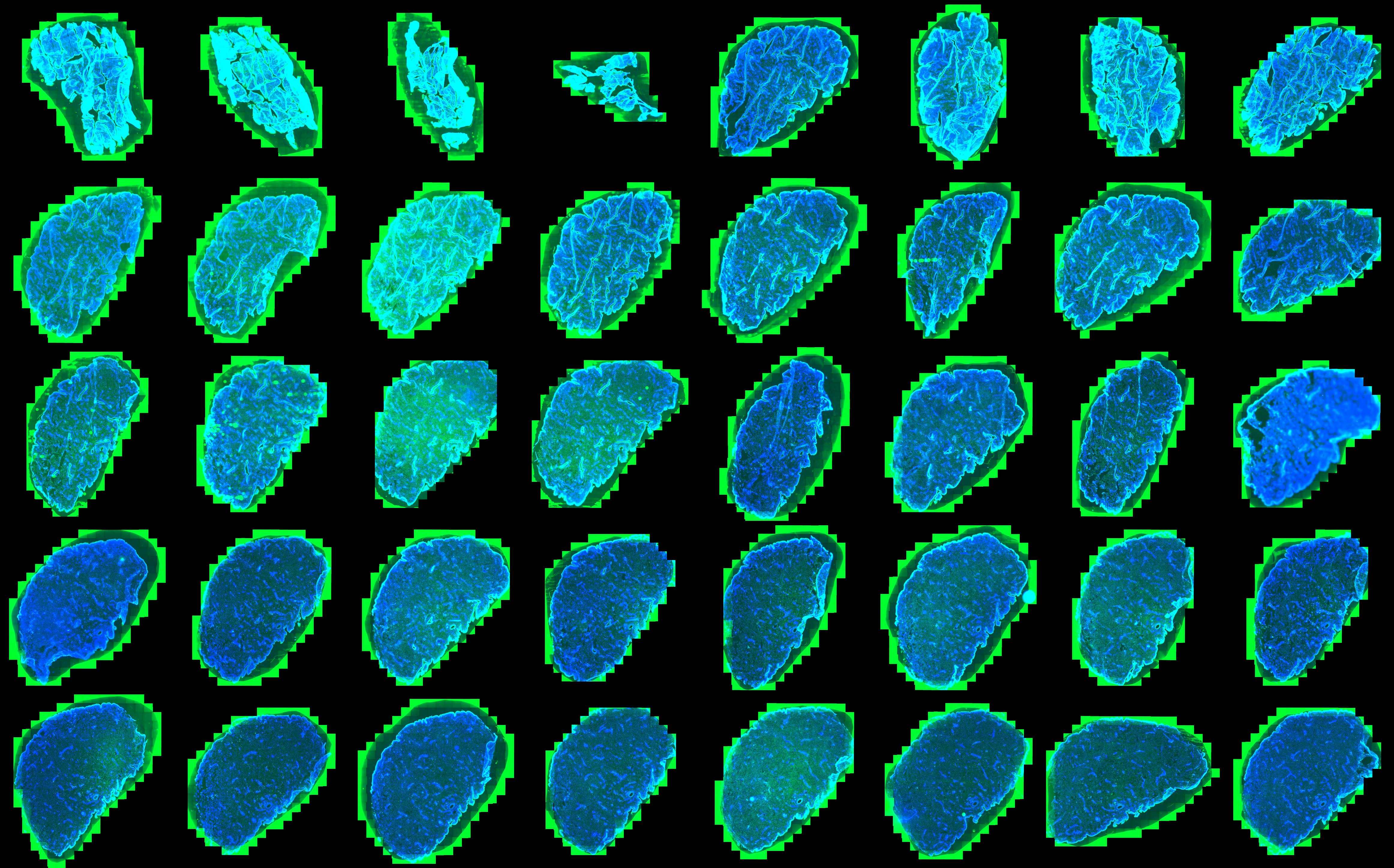

Supplement: Supplementary file 1 [file pharmaceutics-14-00151-s001.zip › File S2 - all samples images/10 - dermojet 1 head - polymer - 24h - GFP.jpg]

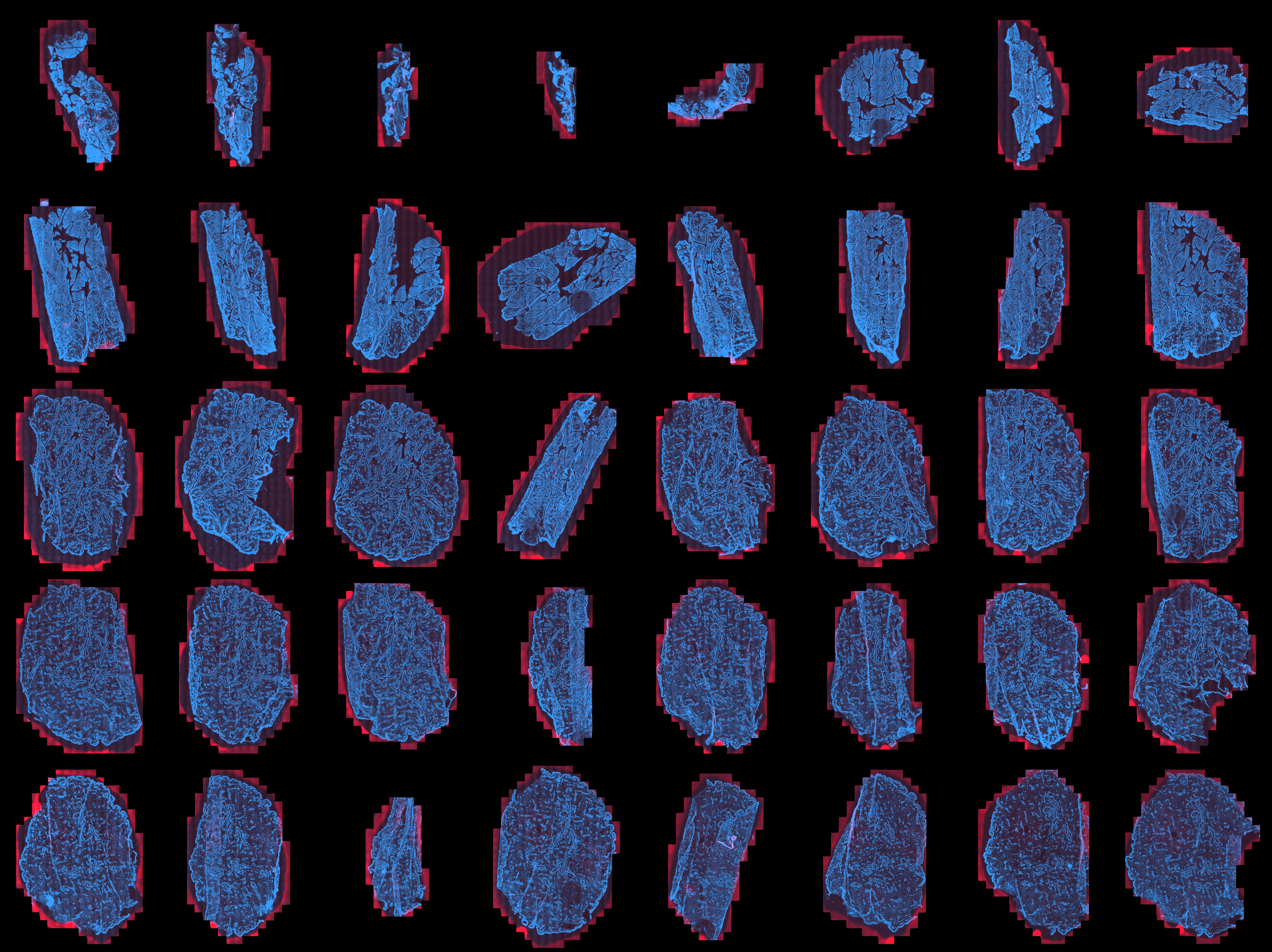

Supplement: Supplementary file 1 [file pharmaceutics-14-00151-s001.zip › File S2 - all samples images/11 - dermojet 3 heads - polymer - 24h - cy5.jpg]

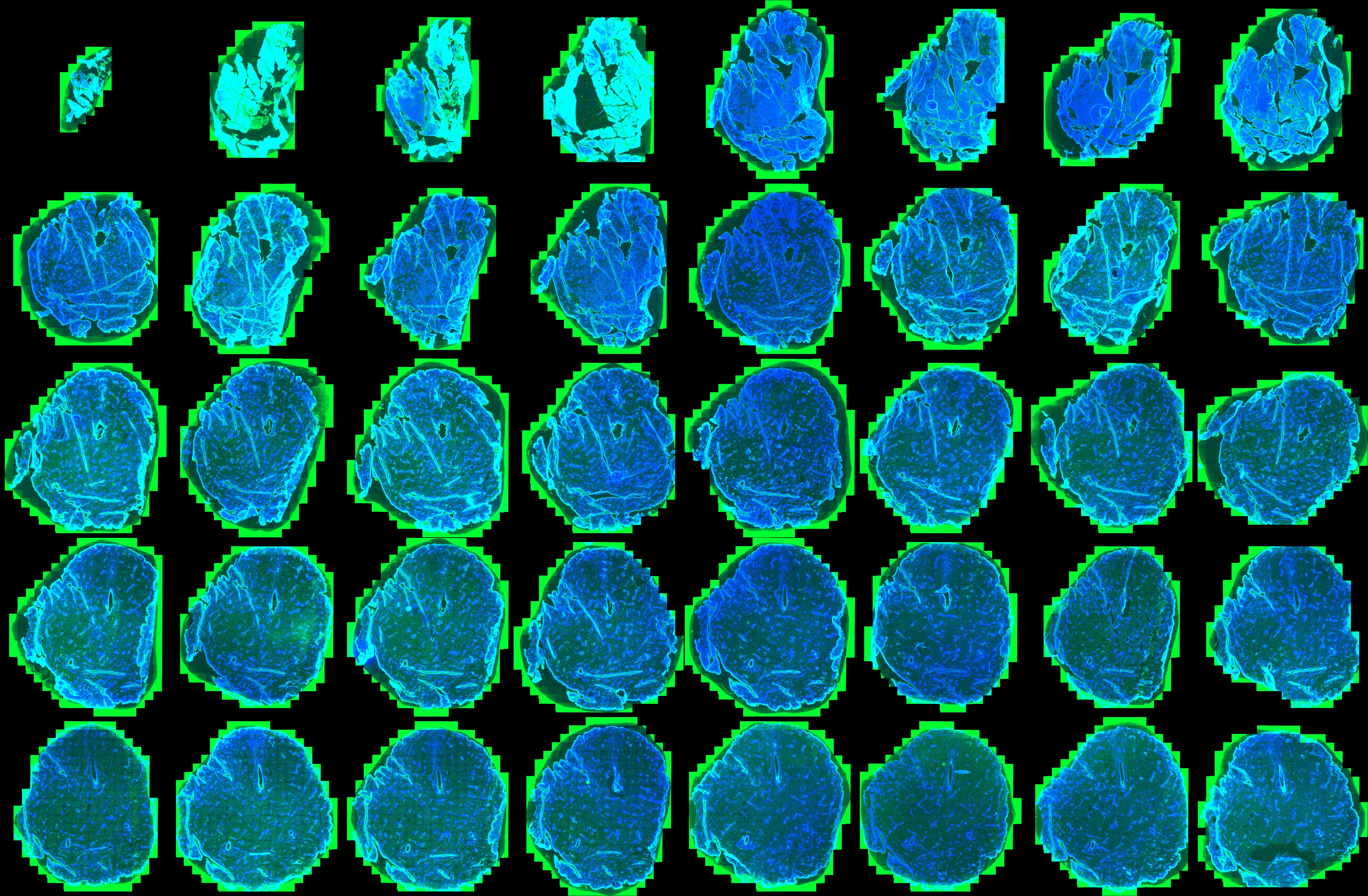

Supplement: Supplementary file 1 [file pharmaceutics-14-00151-s001.zip › File S2 - all samples images/12 - dermojet 3 heads - polymer - 24h - GFP.jpg]

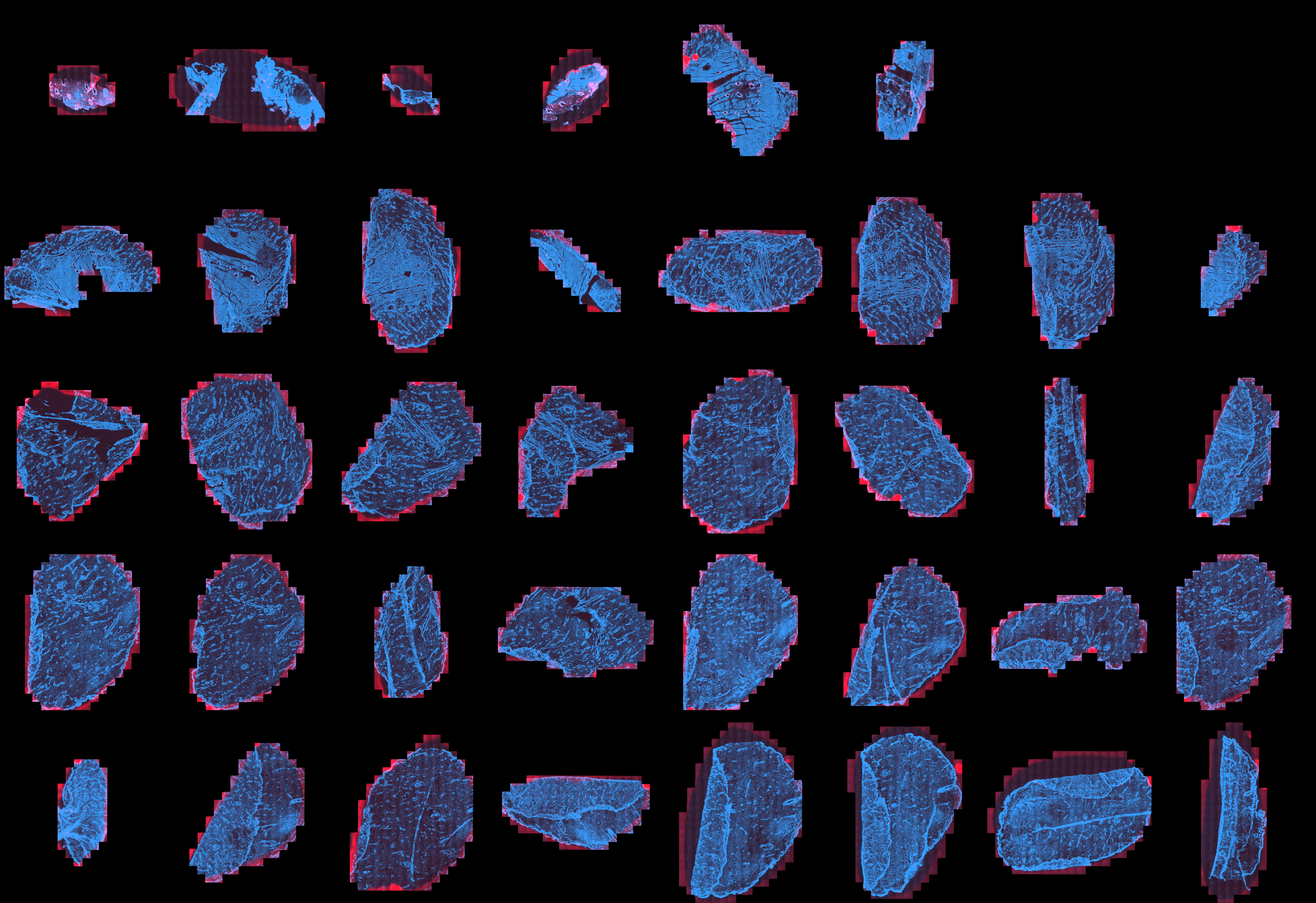

Supplement: Supplementary file 1 [file pharmaceutics-14-00151-s001.zip › File S2 - all samples images/13 - laser poration - polymer - 24h - cy5.jpg]

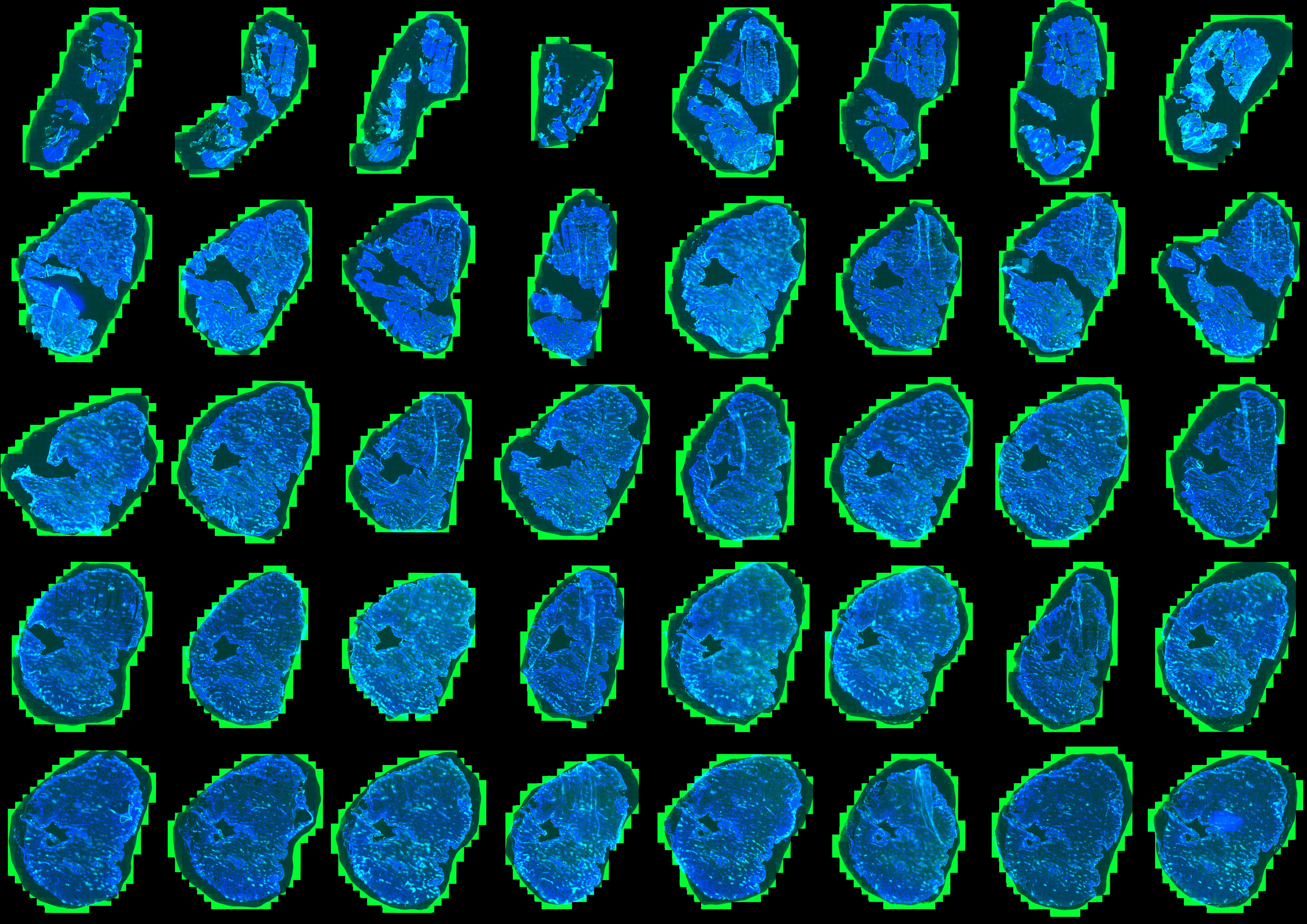

Supplement: Supplementary file 1 [file pharmaceutics-14-00151-s001.zip › File S2 - all samples images/14 - laser poration - polymer - 24h - GFP.jpg]

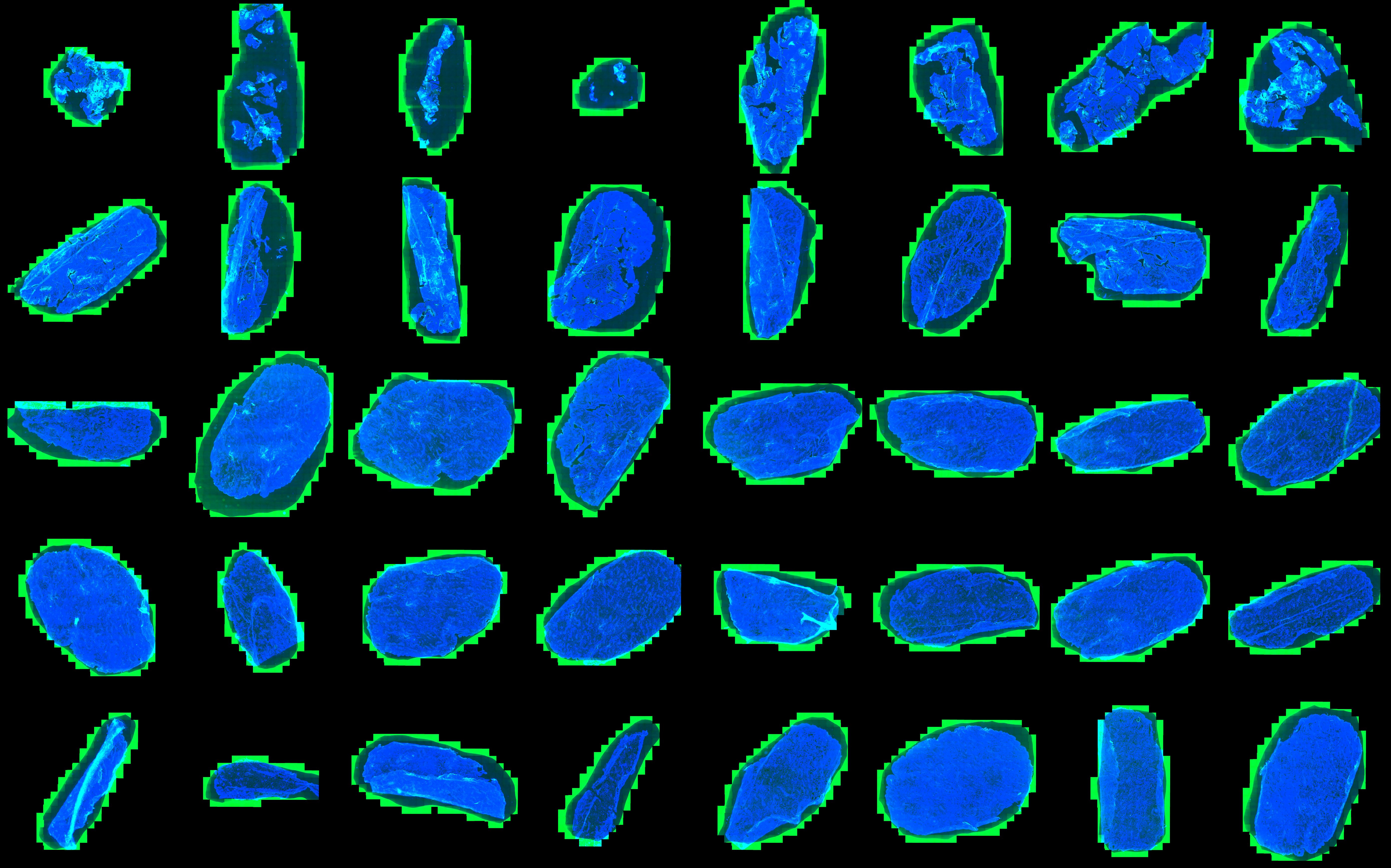

Supplement: Supplementary file 1 [file pharmaceutics-14-00151-s001.zip › File S2 - all samples images/2 - untreated GFP.jpg]

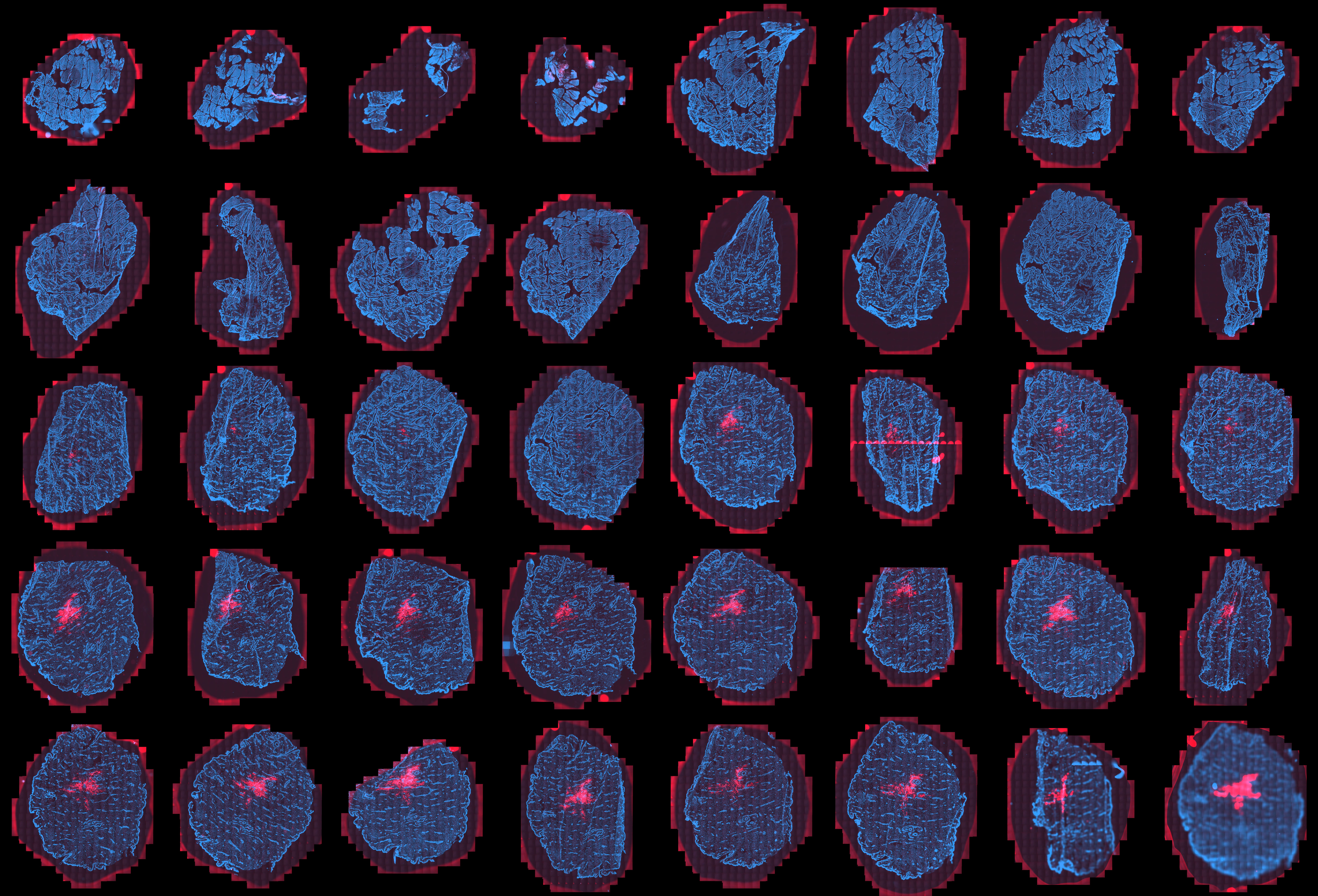

Supplement: Supplementary file 1 [file pharmaceutics-14-00151-s001.zip › File S2 - all samples images/3 - intradermal injection - polymer - 24h - cy5.jpg]

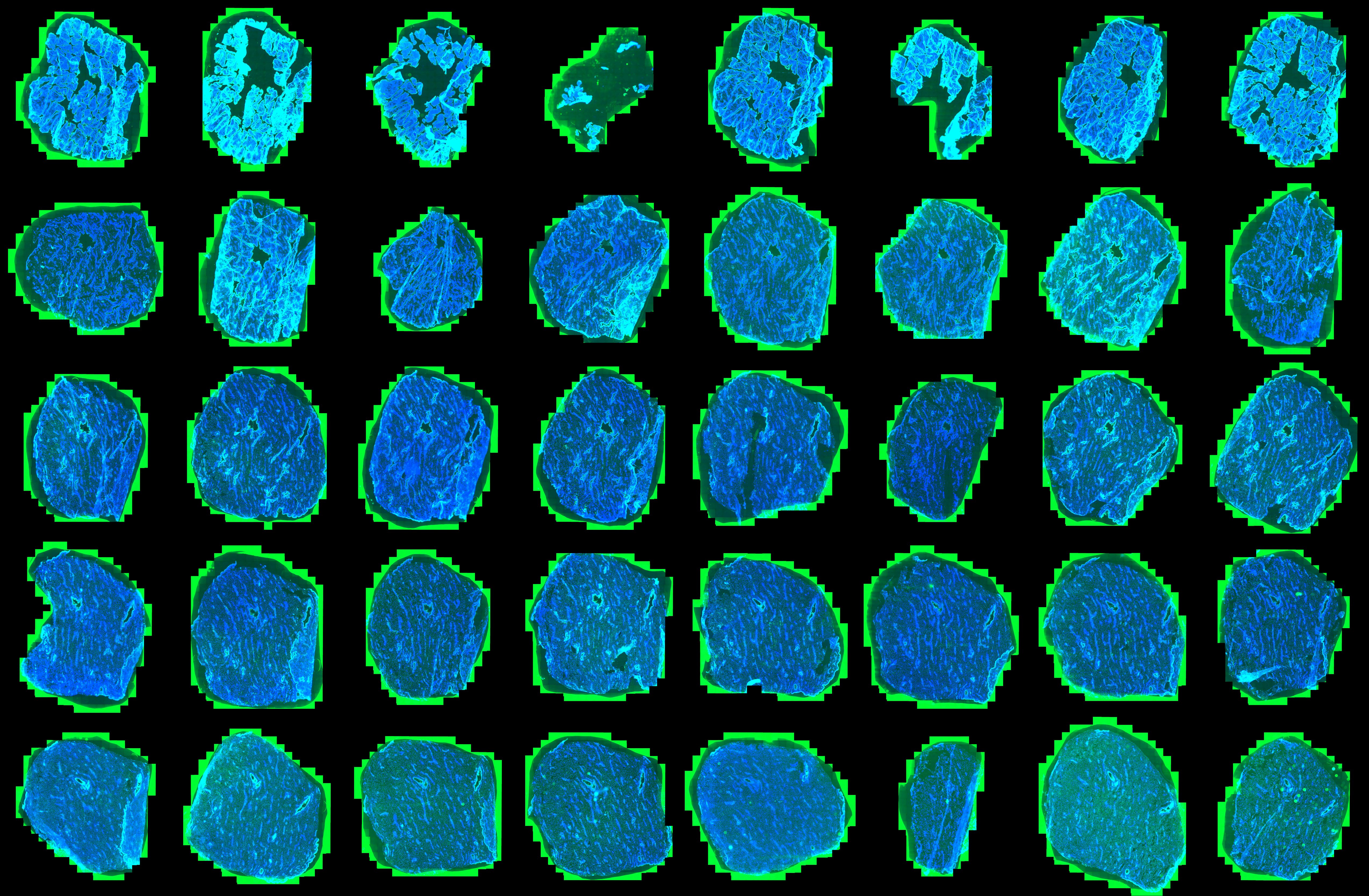

Supplement: Supplementary file 1 [file pharmaceutics-14-00151-s001.zip › File S2 - all samples images/4 - intradermal injection - polymer - 24h - GFP.jpg]

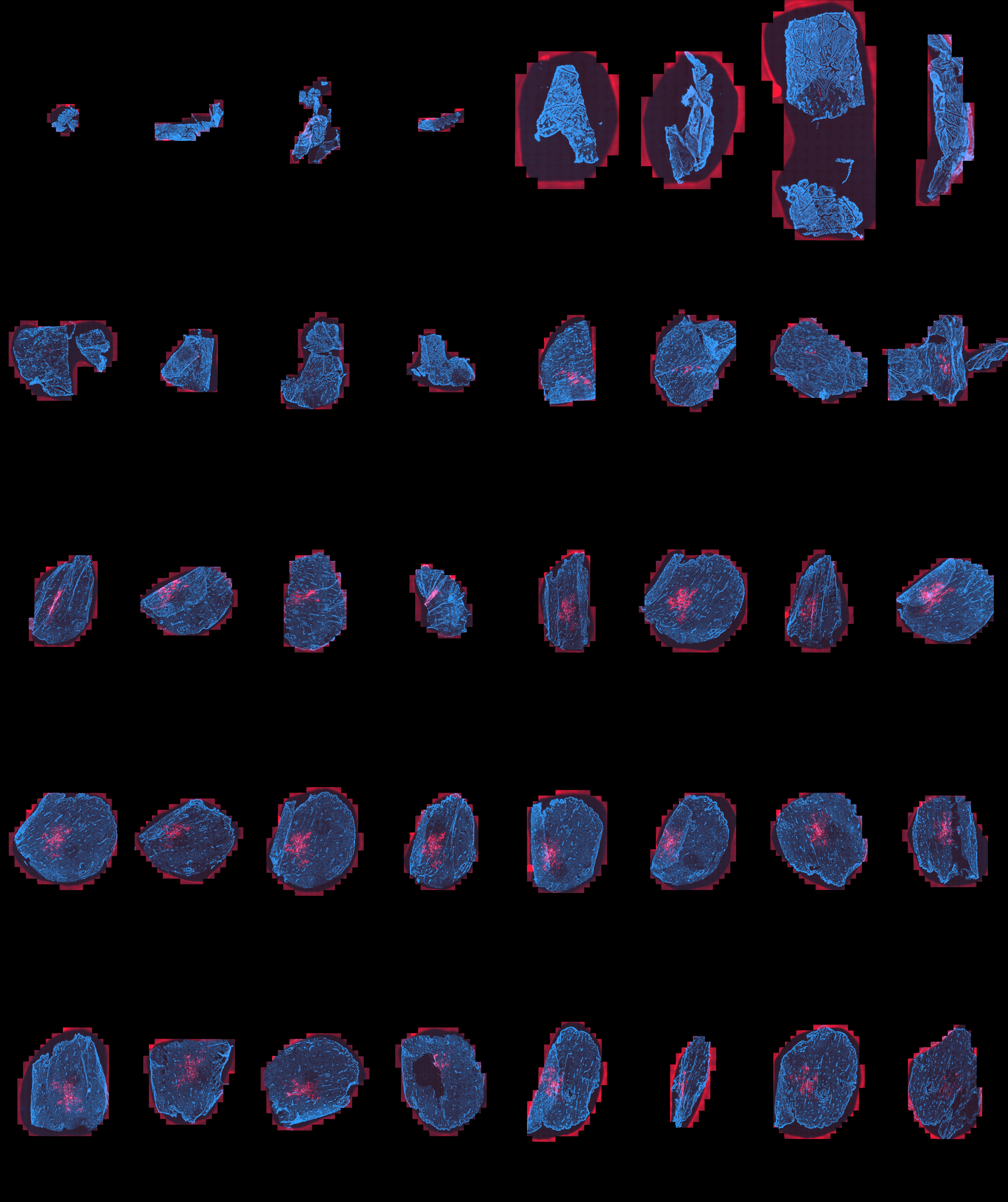

Supplement: Supplementary file 1 [file pharmaceutics-14-00151-s001.zip › File S2 - all samples images/5 - intradermal injection - polymer - 48h - cy5.jpg]

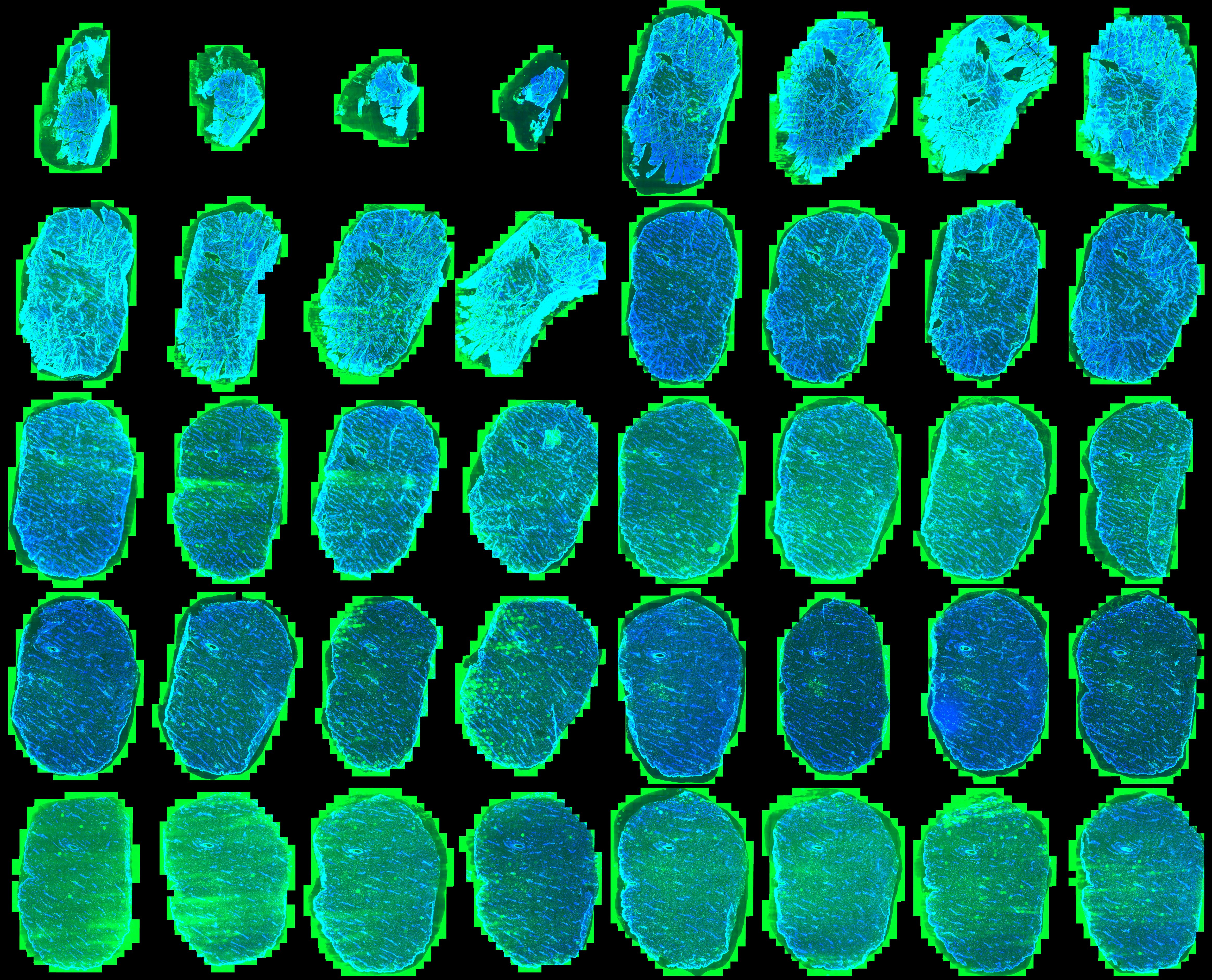

Supplement: Supplementary file 1 [file pharmaceutics-14-00151-s001.zip › File S2 - all samples images/6 - intradermal injection - polymer - 48h - GFP.jpg]

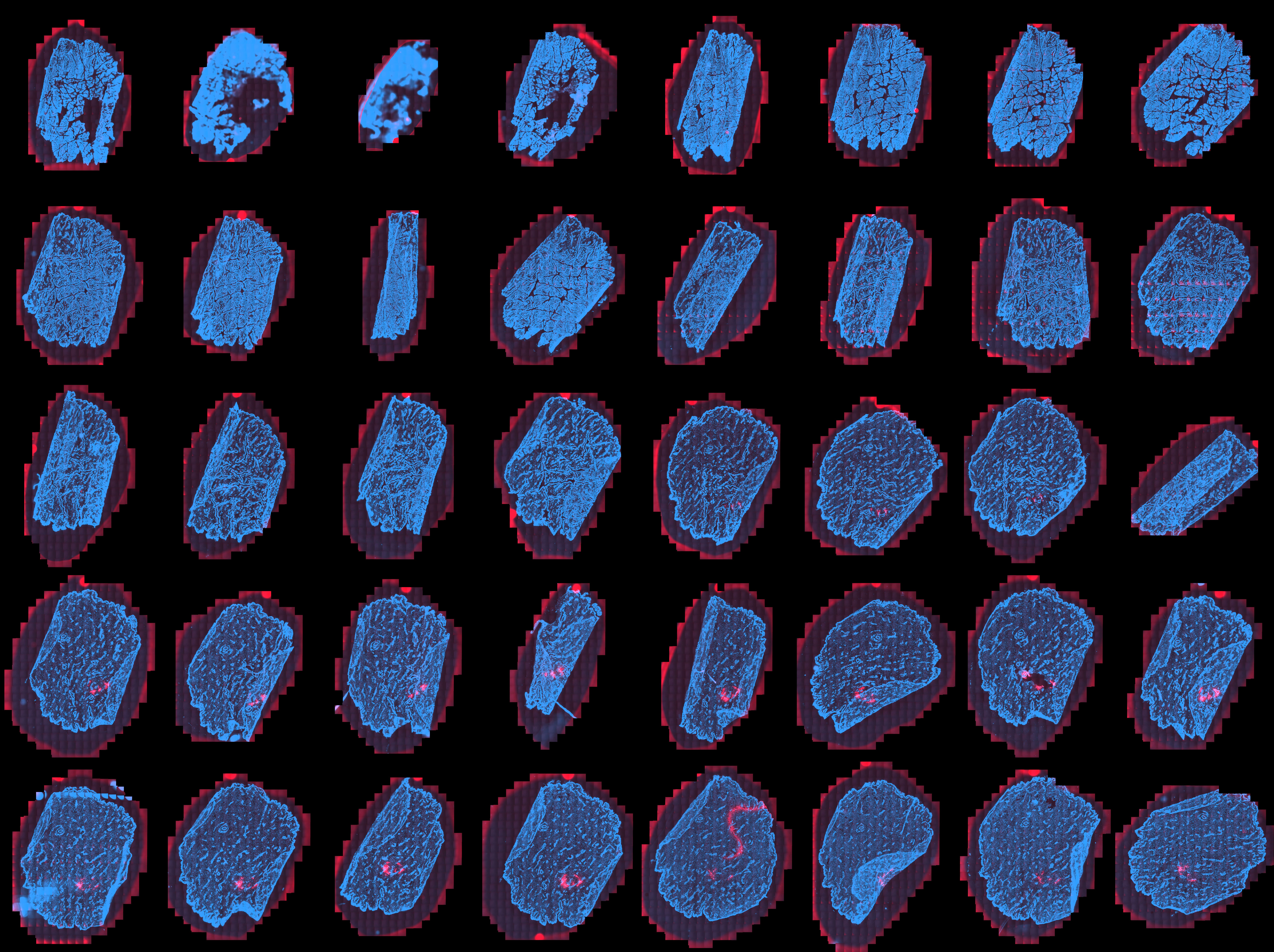

Supplement: Supplementary file 1 [file pharmaceutics-14-00151-s001.zip › File S2 - all samples images/7 - hollow microneedle injection - polymer - 48h -cy5.jpg]

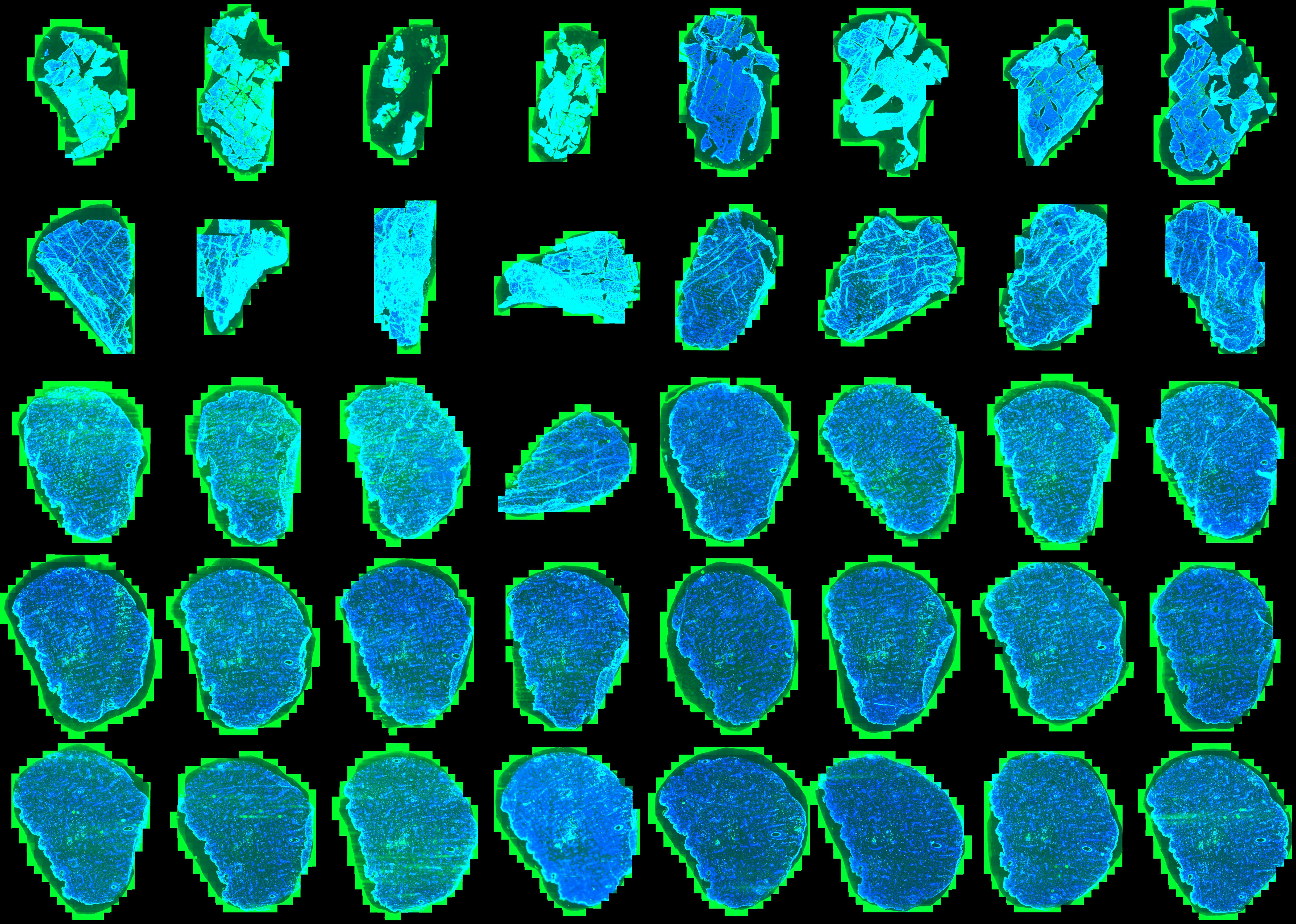

Supplement: Supplementary file 1 [file pharmaceutics-14-00151-s001.zip › File S2 - all samples images/8 - hollow microneedle injection - polymer - 48h - GFP.jpg]

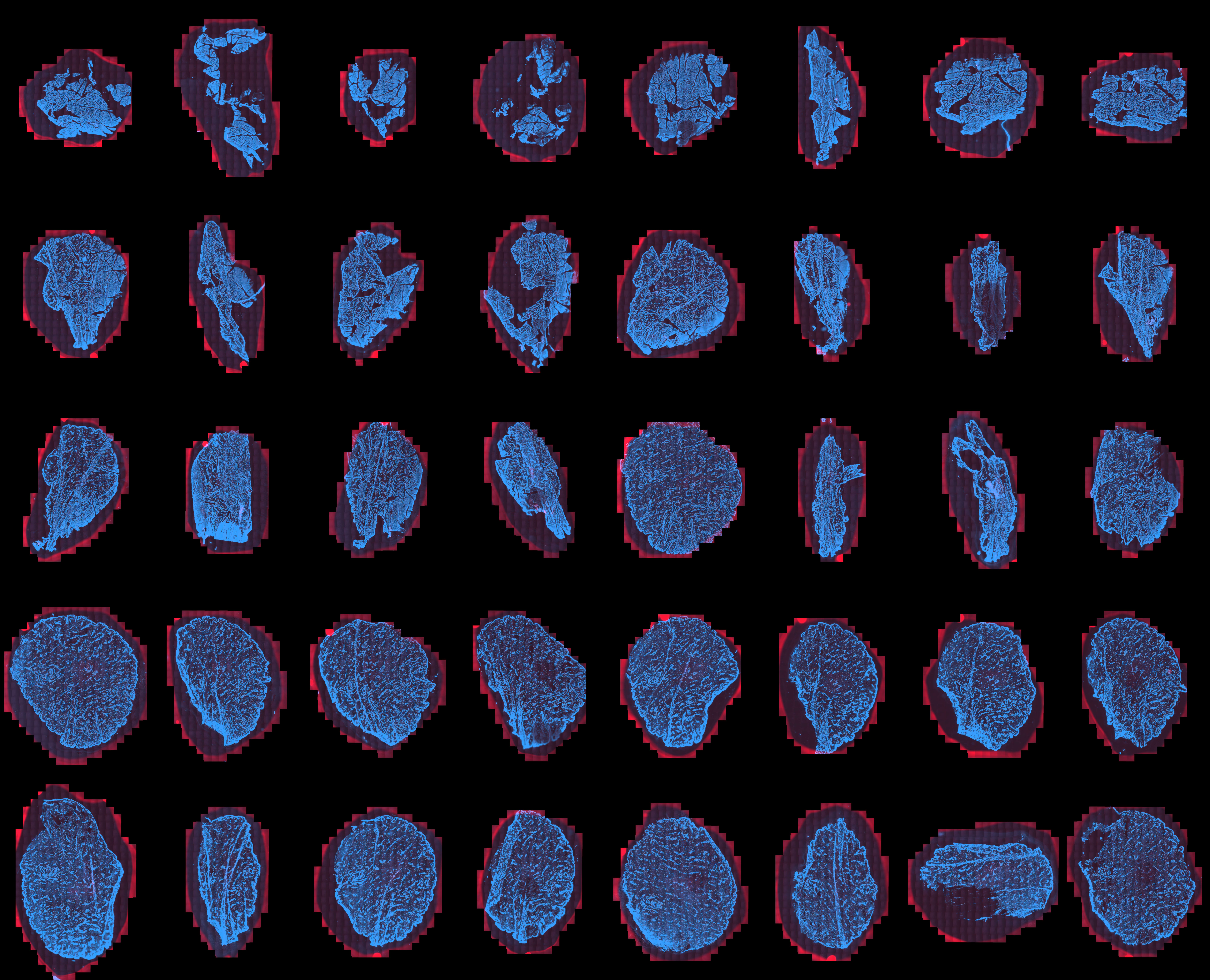

Supplement: Supplementary file 1 [file pharmaceutics-14-00151-s001.zip › File S2 - all samples images/9 - dermojet 1 head - polymer - 24h - cy5.jpg]
